# Exploring the Potential of *Micrococcus luteus* Culture Supernatant With Resuscitation-Promoting Factor for Enhancing the Culturability of Soil Bacteria

**DOI:** 10.3389/fmicb.2021.685263

**Published:** 2021-06-29

**Authors:** Marco Antonio Lopez Marin, Michal Strejcek, Petra Junkova, Jachym Suman, Jiri Santrucek, Ondrej Uhlik

**Affiliations:** Faculty of Food and Biochemical Technology, Department of Biochemistry and Microbiology, University of Chemistry and Technology, Prague, Czechia

**Keywords:** resuscitation-promoting factor, extracellular organic matter, non-cultured bacteria, *Micrococcus luteus*, increased culturability, viable but non-culturable state, oligotrophic medium

## Abstract

A bacterial species is best characterized after its isolation in a pure culture. This is an arduous endeavor for many soil microorganisms, but it can be simplified by several techniques for improving culturability: for example, by using growth-promoting factors. We investigated the potential of a *Micrococcus luteus* culture supernatant containing resuscitation-promoting factor (SRpf) to increase the number and diversity of cultured bacterial taxa from a nutrient-rich compost soil. Phosphate-buffered saline and inactivated SRpf were included as controls. After agitation with SRpf at 28°C for 1 day, the soil suspension was diluted and plated on two different solid, oligotrophic media: tenfold diluted Reasoner’s 2A agar (R2A) and soil extract-based agar (SA). Colonies were collected from the plates to assess the differences in diversity between different treatments and cultivation media. The diversity on both R2A and SA was higher in the SRpf-amended extracts than the controls, but the differences on R2A were higher. Importantly, 51 potentially novel bacterial species were isolated on R2A and SA after SRpf treatment. Diversity in the soil extracts was also determined by high-throughput 16S rRNA amplicon sequencing, which showed an increase in the abundance of specific taxa before their successful cultivation. Conclusively, SRpf can effectively enhance the growth of soil bacterial species, including those hitherto uncultured.

## Introduction

The isolation of microorganisms in pure cultures has remained one of the main pillars of microbiology since its beginnings. More recently, culture-independent methods (e.g., metagenomics) have provided an immense amount of knowledge on bacterial diversity and functions (Escobar-Zepeda et al., 2015) that the relevance of culturing as a microbiological technique looks somewhat dwarfed in comparison. However, culturing is still necessary, and efforts are still made to isolate yet-uncultured bacteria (Ling et al., 2015; Ding et al., 2020). Among other functions, novel isolates can help us better understand the phenotypes of antibiotic-resistant bacteria (McLain et al., 2016), can be a repository of pharmaceutically bioactive compounds (Manivasagan et al., 2014) or can be used to foster biotechnological areas such as bioremediation and bioprospecting (Pascoal et al., 2020). In general, culturing provides a basis upon which to corroborate the immense quantity of data generated by culture-independent sequencing-based approaches (Nichols, 2007). While novel culturing methods can be very diverse (Pham and Kim, 2012; Overmann et al., 2017), several still rely on the use of the petri dish. Because of its versatility, the petri dish is still a central tool for the recent field of culturomics, the “extensive assessment of microbial composition by high-throughput culture” (Greub, 2012; Nowrotek et al., 2019). Richard Petri’s development of a simple device for culturing bacteria has thus withstood the test of time and remains a protean tool for research, even in the genomic era.

Co-culturing is one strategy for increasing culturability. This is either growing a previously uncultured bacterium together with a fully culturable helper cell, or adding signaling components or growth-promoting factors to a culture in which bacteria are growing (Stewart, 2012). Because microorganisms do not live in isolation in their natural environments, but are a part of a complex community, the addition of components produced by other organisms may induce the growth of fastidious bacteria. Signaling components such as siderophores and quorum sensing compounds have been shown to increase the culturability of bacteria *in vitro* (Bruns et al., 2002, 2003; D’Onofrio et al., 2010).

Growth-promoting factors can also increase bacterial culturability by inducing their resuscitation or facilitating their replication (Mukamolova et al., 1999). In their environments, bacteria can subsist dormant as a defense mechanism for coping with deleterious environmental conditions (Mukamolova et al., 2003). Other bacteria, such as taxa belonging to the phylum Acidobacteria, are generally hard to culture despite being widely distributed in the biosphere and have only few described representatives (Dedysh and Yilmaz, 2018). If a bacterium is transported dormant from its environment onto a laboratory medium, it can remain dormant and, thus, unculturable. One of the most widely researched molecules that induces bacterial resuscitation is the resuscitation-promoting factor (Rpf) produced by the bacterium *Micrococcus luteus* (Pinto et al., 2015). Rpf is a member of a broad protein family produced mainly by high GC-content gram-positive bacteria such as Actinobacteria (Mukamolova et al., 2003). Although the enzymatic activity of Rpf is directly linked to its resuscitation potential (Mukamolova et al., 2006), its exact resuscitation mechanism remains unknown (Sexton et al., 2020). Molecules analogous to Rpf extend to other bacterial clades, such as the Firmicutes (Shah and Dworkin, 2010). In addition to Rpf, other signaling molecules, including homoserine lactones, cyclic adenosine monophosphate (Bruns et al., 2002), or metabolites such as zincmethylphyrins and coproporphyrins (Bhuiyan et al., 2016) can also act as growth promoting factors and can be potentially used to enhance the culturability of bacteria.

In recent research, Rpf has been shown to boost biotechnological processes such as the degradation of phenol and other contaminants in wastewater (Jin et al., 2017; Li et al., 2018; Su et al., 2018a, 2019; Yu et al., 2020), polychlorinated biphenyls (Su et al., 2013, 2015, 2021; Ye et al., 2020) and cellulose in soil (Su et al., 2018b). Several previously uncultured bacteria with PCB-degrading capabilities were isolated with the aid of Rpf-containing supernatants, specifically high GC-content gram-positive organisms related to the genera *Rhodococcus* and *Arthrobacter*, and gram-negative bacteria belonging to the family Alcaligenaceae (Su et al., 2013). In addition, the resuscitation potential of Rpf can be used in tandem with low-nutrient media that resemble the oligotrophic conditions encountered by bacteria in natural environments (Kaprelyants et al., 1993).

**FIGURE 1:**
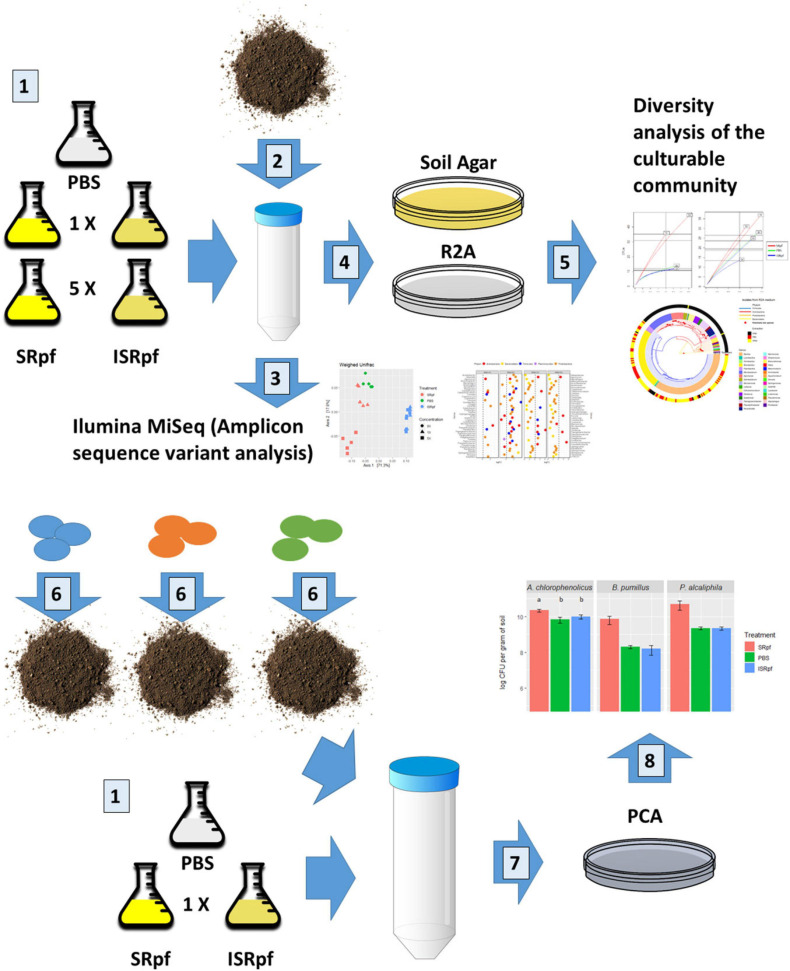
Summary of the experimental design. Number 1: *M. luteus* was cultured in lactate minimal medium. A concentration 5× was obtained by culturing a 5× larger volume and concentrating it to the volume of 1×. Both concentrations were divided in two and one half was inactivated. Supernatant components of the *M. luteus* culture larger than 3 kDa were then washed with PBS. A PBS only extraction was used as control. Number 2: 1 g of soil was mixed with 9 ml of either of the 5 extraction media. Soil suspensions were incubated for 1 day at 28°C. Number 3: DNA from the soil suspensions was extracted and 16S rRNA sequences amplified (amplicon sequence variants analysis). Number 4: soil suspensions of each extraction strategy (number 1) were serially diluted and plated on two different solid media. Number 5: colonies were selected from the plates and dereplicated with MALDI TOF-MS and their 16S rRNA genes were partially sequenced. For the bioaugmentation experiment (number 6), three different bacteria were inoculated in the sterile soil and incubated for a month and extracted as described in number 1 (just 1× concentration). Number 7: the soil suspensions with the inoculated bacteria were serially diluted and plated on PCA (drop method). Number 8: colonies were counted for each drop.

In this work, we analyzed the potential of a *Micrococcus luteus* supernatant containing Rpf (SRpf) which was added to soil during the extraction of microorganisms to increase the culturability of soil bacteria on two different oligotrophic media. Because we worked under the hypothesis that SRpf’s enzymatic activity is responsible for an increased culturability, inactive SRpf (ISRpf) was included as a control, acting as a potential extra carbon source. The effects of SRpf and ISRpf were compared to an extraction using only phosphate-buffered saline (PBS), which serves as an additional control. After the extraction with SRpf or the controls (ISRpf and PBS), the soil suspension was serially diluted and plated on two low-nutrient media: a medium composed of soil extracts (SA) and 1/10 diluted Reasoner’s 2A medium (R2A). The same extraction technique was used to assess culturability differences with selected bacterial strains inoculated in sterile soil and incubated for longer periods of time (1 month) without any substrate addition. The reaction of soil bacteria to SRpf was assessed by community changes in soil suspensions through 16S rRNA amplicon sequencing, while the diversity on plates was assessed by 16S rRNA gene sequencing after dereplication with MALDI-TOF MS (Figure 1).

## Materials and Methods

### *Micrococcus luteus* Cultivation and Protein Fraction Purification

*Micrococcus luteus* NCTC 2665 (purchased from DSMZ, Germany) was inoculated in lactate minimal medium (Su et al., 2014). The medium composition was (per liter): 8.75 g lithium lactate, 0.005 g biotin, 0.02 g L-methionine, 0.04 g thiamine, 1 g inosine (all Sigma Aldrich, United States); 4 g NH_4_Cl, 1.4 g KH_2_PO_4_, 0.03 g MgSO_4_ (all Penta, Czechia). A trace element solution of the following composition (per liter) was added to the medium (1.5 milliliters per liter): 0.375 g CuSO_4_⋅5H_2_O, 0.785 g MnCl_2_⋅4H_2_O, 0.029 g Na_2_MoO_4_⋅2H_2_O, 0.089 g ZnSO_4_⋅7H_2_O (all Lachema, Czechia); and 0.183 g FeSO_4_⋅7H_2_O (Lachner, Czechia). The growth of the culture was monitored by absorbance at 600 nm (Abs_600_) in a spectrophotometer (Beckman Coulter, Brea, CA, United States). When an Abs_600_ of 2.9 was reached (point of maximum protein content), the cells were separated from the supernatant by centrifugation at 5,000 × *g* for 10 min, and thereafter the supernatant was filtered through a 0.22 μm membrane (Steritop^TM^ Filter Units, Merck Millipore, Germany). The sterile supernatant was then ultrafiltered using a 3 kDa Amicon^®^ Ultra 15 ml centrifugal filter (Merck Millipore Ltd., Cork, Ireland) to concentrate the protein content. Half of the volume of this high-protein-content supernatant was autoclaved to inactivate its enzymatic activity. The other half was refrigerated until used (for no longer than 1 day at 4°C).

After autoclaving, the high-protein-content supernatant was also chemically inactivated. Dithiothreitol (DTT) was added to SRpf to a concentration of 5 mM and incubated for 45 min at 56°C to reduce thioester bonds. Iodoacetamide was added thereafter at a concentration of 25 mM to alkylate SH groups and incubated for 30 min at 25°C in the dark. Both volumes of supernatant (active and inactive) were washed five times with phosphate-buffered saline (PBS) using the same 3 kDa centrifugal recovery units to remove the residual lactate and other low molecular weight substances. After washing, the concentrated protein fractions were diluted in PBS to the volume of the original *M. luteus* culture (1×) and its fivefold concentrated equivalent (5×). Here, these protein fractions in PBS are called SRpf (supernatant containing Rpf) and ISRpf (its inactive version).

### Determination of Total Protein Concentration in the Supernatant and Rpf Detection

The protein content of the growing *M. luteus* culture in lactate minimal medium was determined in a periodically sampled small aliquot of culture (5 ml). Cells were separated off the supernatant by centrifugation and filtration as previously described, and the sterile aliquots were concentrated by lyophilization. Briefly, the sterile aliquots were frozen (−80°C) and placed in a lyophylizator (Labconco Corporation, United States) at −50°C and 0.03 mBar until the ice evaporated. Protein concentration in these samples was determined according to Popov (Popov et al., 1975). The maximum protein content in the supernatant occurred at an Abs_600_ of 2.9. A similar behavior of the Rpf production has been previously reported (Telkov et al., 2006).

Resuscitation-promoting factor in the supernatant was detected by sodium dodecyl sulfate polyacrylamide gel electrophoresis (SDS Page) in tandem with mass spectrometry. A fraction of the lyophilized aliquot extracted from the *M. luteus* culture was mixed with staining buffer according to Laemmli (1970). Gel bands were cut into 1 mm cubes. The cubes were distained using a mixture of acetonitrile and 0.1 M ammonium bicarbonate in a 1:1 ratio. The distained cubes were dried with acetonitrile, and a solution of DTT was added to reduce disulfide bonds. The reduction was carried out at 56°C for 45 min. Thereafter, alkylation was carried out with iodoacetamide in the dark and at room temperature for 30 min. The cubes were then washed with an acetonitrile/ammonium bicarbonate solution for 15 min and dried again with acetonitrile. A solution of trypsin (12.5 ng/μl) was added to the cubes and incubated at 4°C for 25 min. The trypsin excess was removed, and a fresh solution of 50 mM ammonium bicarbonate was added to cover the gel cubes. The digestion with trypsin was carried out for 6 h at 37°C, and then stopped with trifluoroacetic acid (TFA). The peptide-containing solution was pipetted to a clean tube, and cubes were soaked with 0.1% TFA solution. The cubes were sonicated for 15 min in order to release the peptides left inside the cubes. Both peptide-containing volumes were combined after sonication, cleaned up with ZipTip and left to evaporate.

The mass spectrometric analysis was performed with a UHPLC Dionex Ultimate3000 RSLC nano (Dionex, Germany) coupled to an ESI-Q-TOF Maxis Impact mass spectrometer (Bruker Daltonics, Germany). Dried samples were dissolved in a mixture of water:acetonitrile:formic acid (97:3:0.1%) and loaded into the trap column, an Acclaim PepMap 100 C18 (100 μm × 2 cm, particle size 5 μm, Dionex, Germany), with a mobile-phase flow rate of 5 μL/min of A (0.1% formic acid in water) for 5 min. The peptides were then separated in the analytical column, an Acclaim PepMap RSLC C18 (75 μm × 150 mm, particle size 2 μm, Dionex, Germany), and eluted with mobile-phase B (0.1% formic acid in acetonitrile) using the following gradient: 0 min 3% B, 5 min 3% B, 35 min 35% B, 32 min 90% B, 50 min 90% B, 51 min 3% B, and 60 min 3% B. The flow rate during the gradient separation was set to 0.3 μL/min. Peptides were eluted directly to the ESI source-captive spray (Bruker Daltonics, Germany). Measurements were performed in DDA mode with precursor-ion selection in the range of 400–1,400 Da. Up to 10 precursor ions were selected for fragmentation from each MS spectrum.

Peak lists were extracted from the raw data with the software Data Analysis 4.1 (Bruker Daltonics, Germany). Proteins were identified in the software Proteinscape 3.1 (Bruker Daltonics, Germany) using in-house Mascot server 2.4.1 (Matrix Science) with the SwissProt protein database downloaded from http://www.uniprot.org (January 2014). The parameters for the database search were set as follows: carbamidomethyl (C) as fixed modification, oxidation (M) and as variable modifications, tolerance 10 ppm in MS mode and 0.05 Da in MS/MS mode, enzyme trypsin 1 miscleavage. Only proteins with a Mascot score of at least 30 were accepted.

### SRpf Activity Determination

Supernatant activity was determined fluorometrically, similarly to the procedure by Telkov et al. (2006). The artificial substrate 4-methylumbelliferyl-beta-D-N,N′,N″-triacetylchitotrioside (MUF tri-NAG) was added to a final concentration of 800 μM to either 100 μl PBS, 100 μl SRpf or 100 μl ISRpf. The supernatants, the PBS without added substrate and the substrate alone all served as blanks for the MUF tri-NAG cleavage reaction. The reaction was monitored for 7 h at 28°C in a Fluoroskan Ascent FL microplate fluorometer and luminometer with one dispenser (Thermo Fisher Scientific Oy, Finland) with an excitation wavelength of 355 nm. Fluorescence units were converted to mass units by a standard calibration curve constructed from four measurements of 4-methylumbelliferone diluted in methanol (the fluorescent product of the MUF tri-NAG cleavage), covering a mass between 0 and 0.1 mg.

### Bioaugmentation of Sterile Soils With Selected Bacterial Strains

Soil was collected from a homemade garden compost in Mirošovice, central Bohemia, Czechia. The garden compost was made by the constant piling of organic matter such as grass, and fruit and vegetable remains between the spring and fall of 2016. The soil was refrigerated before use (for 3 years) to induce microbial dormancy. The soil was a sandy loam with a smooth texture. Its composition was analyzed commercially (ALS Czech Republic): its dry matter content was 90.3%, organic matter content 23.6%, pH 7.2, the percentage of particles 2–4 mm was 0.35%; 1.37% bigger particles and 12.1% clay particles. The soil was sieved through a 1 mm filter before use, but was not sieved for the preparation of the soil agar (see below).

The model bacterial strains *Pseudomonas alcaliphila* JAB1 (Ridl et al., 2018), *Bacillus pumilus* SAFR032 (Link et al., 2004) and *Arthrobacter chlorophenolicus* A6 (Westerberg et al., 2000) were grown in half-strength lysogeny broth (LB) until they reached an Abs_600_ of 1. The cultures were centrifuged (5,000 × *g* for 10 min) and the pellet was washed in 0.85% NaCl to remove the LB, after which 1 ml of the Abs_600_ 1 culture was inoculated in 5 g of sterile soil in sterile 50 mL tubes. The soil was sterilized at 180°C for 3 h. This procedure was repeated several times on successive days until no growth was observed on plate count agar (PCA). Each strain was inoculated in nine tubes (in triplicates, for the extraction with SRpf, ISRpf and PBS). The samples were incubated at 28°C for 1 month.

### SA and Diluted R2A Preparation

The soil used for the extraction of soil bacteria was also used for preparing the SA. The preparation of the SA was similar to the preparation described by Hamaki et al. (2005). The soil was suspended in distilled water at a concentration of 10% w/v (weighed by dry matter). The suspension was agitated for 1 day at 28°C. The dissolved soil fraction was decanted, centrifuged at 5,000 × *g* for 10 min, and filter-sterilized through a 0.22 μm membrane. Agar was added to this sterile fraction in a ratio of 2:9 by volume (agar:sterile soil fraction). The agar content per liter of SA was 14 g. R2A (Himedia Laboratories, India) was diluted 10 times in distilled water. Extra agar was added to reach a concentration of 14 g l^–1^.

### Extraction of Bacteria From Soil and Culturing

Nine ml of SRpf and ISRpf, 1× and 5× concentrated, were mixed with 1 g of the non-sterile compost soil in 50 mL tubes. Each combination consisted of five biological replicates (20 tubes in total). Five more replicates were extracted only with PBS (control). The 25 tubes were agitated for 24 h at 120 rpm and 28°C. Thereafter, the soil suspensions were serially diluted and plated either on diluted R2A or SA and incubated for 1 week at 28°C. The dilution that formed less than 200 colonies per plate was selected for colony picking (one plate selected per tube). This number of CFUs was achieved when soil suspensions were serially diluted in 1:10 steps until suspensions were diluted a million times. From each selected plate, on average 13 (SD 5) colonies were randomly collected. The randomness was ensured by numbering the colonies prior to selection and X random numbers were then picked using a random number generator. Collected colonies were transferred individually to another plate with the same medium, where they were cultured for 3 days before the recurrent isolate dereplication using MALDI-TOF MS and 16S rRNA gene sequencing (see further).

Bioaugmented sterile soils were extracted in the same way, but in this case 30 ml of 1× SRpf, 1× ISRpf or PBS were used per tube. The serially diluted suspensions (10 fold dilutions) were then plated on PCA plates using the drop plate method (Herigstad et al., 2001). Briefly, for each tube and each dilution, five drops were applied on PCA. Plates were cultured at 28°C for 3 days before CFU counting. CFUs were counted for each drop (15 drops per treatment and dilution) to obtain the mean and the standard deviation.

### Dereplication of Isolates With MALDI-TOF MS and Their 16S rRNA Gene Sequencing

To reduce the extensive number of isolates retrieved (672 individual colonies growing on plates were isolated), the initial analysis of the diversity of the culturable fraction, both on R2A and SA, was performed with an Autoflex speed MALDI-TOF mass spectrometer (Bruker Daltonics, Germany) as described elsewhere (Strejcek et al., 2018). Briefly, bacterial samples were prepared from the fresh R2A and SA plates using the direct transfer procedure and mixed with α-cyano-4-hydroxy-cinnamic acid (HCCA) matrix (Bruker Daltonics, Germany). Spectra were measured automatically with real-time classification software (Bruker Daltonics, Germany). Spectral processing and clustering were done in R project (R Core Team, 2020) following an in-house R script (Strejcek et al., 2018) with the use of the MALDIquant and MALDIforeign packages (Gibb and Strimmer, 2012; Strejcek et al., 2018). Briefly, mass-spectral taxonomic units (MTUs) approximating bacterial species were created by clustering the mass spectra using the 0.9 spectral similarity threshold. Isolates within the same MTU were assigned a 16S rRNA gene sequence of at least one selected representative isolate (see below for more information on validation of this method). In some cases, mass spectra were not measurable due to the nature of the organism. Isolates from the SA medium were more often difficult to analyze and so were grown on another medium. Such isolates were directly analyzed by 16S rRNA gene sequencing (see data availability for the Genebank codes of all the 16S rRNA sequences).

DNA from the colonies was isolated by using simple thermal lysis as described by Wald et al. (2015), or, if unsuccessful, with the use of a PureLink^TM^ Genomic DNA Mini Kit (INVITROGEN, Carlsbad, CA, United States) following the manufacturer’s instructions. A nearly complete 16S rRNA gene was amplified with the universal prokaryotic 16S rRNA gene primers: 8 forward (5′-AGAGTTTGATCMTGGCTCAG-3′) and 1509 reverse (5′-GYTACCTTGTTACGACTT-3′) (Lane, 1991) in a volume of 15 μL containing: 7.5 μL KAPA HiFi HotStart ReadyMix (Kapa Biosystems, Boston, MA, United States); 0.3 μM of each primer (Sigma-Aldrich, United States); and 1 μL of lysed cell supernatant or the kit-extracted DNA. The cycling program was set as follows: 5 min at 95°C, 24 cycles of 20 s at 98°C, 15 s at 56°C, 40 s at 72°C and a final extension of 5 min at 72°C. PCR products were sequenced through Sanger capillary sequencing using the universal prokaryotic primer 926 reverse (5′-CCGYCAATTYMTTTRAGTTT-3′) (Walters et al., 2016). The sequences were classified using the assignTaxonomy() function (Wang et al., 2007) implemented in the DADA2 package that was trained on the SILVA reference database (Quast et al., 2012).

From each MTU, at least 1 representative (isolate) was randomly picked and a portion of its 16S rRNA gene was sequenced. That sequence was assigned to the rest of the isolates belonging to the same MTU. Ten different experimental conditions used in this study (three extraction strategies and two different media used for cultivation, plus two extractions with increased SRpf and ISRpf concentration on two solid media) generated in total 480 isolates for which a partial 16S rRNA sequence could be ascribed. These 480 isolates (each one with one 16S rRNA sequence) constituted the basis upon which subsequent diversity analyses were done. In order to validate the dereplication of isolates with MALDI-TOF MS, for some MTUs at least two isolates were selected and their sequences were then aligned and visualized using the software MEGA X (Kumar et al., 2018).

### DNA Isolation From Soil and 16S rRNA Gene High-Throughput Amplicon Sequencing

Total genomic DNA was isolated from the soil suspensions agitated for 1 day with either SRpf, ISRpf or PBS (25 tubes). DNA extraction was performed using the FastDNA Spin Kit for Soil (MP Bio, Solon, OH, United States) according to the manufacturer’s instructions. The universal prokaryotic primers 515 forward (5′-GTGYCAGCMGCNGCGG-3′) and 926 reverse (5′-CCGYCAATTYMTTTRAGTTT-3′) (Fraraccio et al., 2017) were used to target the hypervariable regions V4–V5 of the 16S rRNA genes. The volume of the PCR was 15 μL (with previously described composition). The program was set as follows: 5 min at 95°C, 20 cycles of 20 s at 98°C, 15 s at 56°C, 15 s at 72°C and a final extension of 5 min at 72°C. A volume of 0.5 μL of the PCR product was used as the template for another round of PCR, which was performed under the same conditions except that the final reaction volume was 25 μL, with a primer concentration of 1 μM (for each primer), and 10 cycles. The forward and reverse primers used for the second PCR were modified with sequencing adapters and internal barcodes of variable length (5–8 bp) using the TaggiMatrix spreadsheet courtesy of Travis C. Glenn at the University of Georgia^1^. The resultant PCR products were purified using AMPure XP Beads (Agencourt, Beckman Coulter, United States). Further amplicon-sample library preparation and sequencing analysis in an Illumina MiSeq instrument were performed at the Core Facility for Nucleic Acid Analysis at the University of Alaska Fairbanks, Fairbanks, AK, United States. The whole experimental design is summarized in Figure 1.

### Data Analysis

High-throughput amplicon sequencing data were analyzed using DADA2 (Callahan et al., 2016). A mock community consisting of 15 bacterial strains was included as a positive control and amplified together with the samples (Strejcek et al., 2018). Primer sequences were trimmed off prior to the analysis. The sequences for each biological replicate were trimmed and filtered by their quality (truncLen = c(240,160), maxN = 0, maxEE = c(2,2), truncQ = 2). After dereplication, sequencing errors were removed (DADA2-based removal), denoised forward and reverse reads were merged, and chimeric sequences were removed. This preprocessing yielded between 24,907 and 110,171 reads per sample. To remove low-abundance, noisy data, amplicon sequence variants (ASVs) with abundances less than 40 were discarded. Taxonomy was assigned using the Silva reference database (Quast et al., 2012).

Sequence analysis was carried out in R (R Core Team, 2020) using the Phyloseq package (McMurdie and Holmes, 2013). ASVs and isolate sequences were aligned individually using the package DECIPHER (Wright, 2016). For the ASV analysis, the weighed UniFrac distance (Lozupone and Knight, 2005) was used to evaluate the effects of each treatment and its concentration levels on the communities using permutational analysis of variance (PERMANOVA) implemented in the *adonis* function of the VEGAN package (Oksanen et al., 2019). Five treatments were considered for this analysis (each treatment with a different concentration, SRpf 1× and 5×, ISRpf 1× and 5×, and PBS). *Post hoc* PERMANOVA tests were performed in R using the pairwise Adonis function (Martinez Arbizu, 2020) and FDR correction (Benjamini and Hochberg, 1995). Possible dispersion differences among the five different treatments were determined by the permutation test for homogeneity of multivariate dispersions (betadisper function, Vegan R package) (Oksanen et al., 2019). Sequence data were merged at the genus level before performing the differential abundance analysis using the edgeR package (Robinson et al., 2009). A quasi-likelihood negative binomial (NB) generalized log-linear model was fitted to the data using the function glmQLFit(). Differential expression was determined by comparing each extraction condition (SRpf and ISRpf) and their concentrations individually to the PBS-only extraction. Robust = TRUE was set when estimating the NB dispersion [estimateDisp()]. A false discovery rate cutoff of 0.001 was selected to determine the statistical significance of each taxon. The diversity of the microbial community was described by the number of ASVs observed (after rarefaction of the data to an even depth of 24,522 sequences), the Simpson and the Shannon indexes (Shannon, 1948; Simpson, 1949; Hill et al., 2003).

For the isolate sequences, in order to dereplicate them, hamming distances among the 480 isolates after alignment (culturable fraction data) were calculated using the package Phangorn (Schliep, 2010). These distances were then used to cluster the sequences based on their similarity using the function hclust() (R Core Team, 2020). The resulting clustering was divided using the cutree() function, using cutoff = 0. This analysis yielded 189 different operational taxonomic units (OTUs, see data availability). To determine potentially novel bacterial species, the similarity of each of these 189 OTUs was compared to the closest described type strain using the EzBioCloud 16S rRNA gene database (Yoon et al., 2017). A 16S rRNA gene sequence similarity of 98.65% or lower was considered to be the threshold for defining a potentially novel species (Kim et al., 2014). The diversity of the 480 isolates (isolates from R2A and SA plates) was assessed with the Chao1 indicator (Chao, 1984) using the package rareNMtests (Cayuela and Gotelli, 2014). Isolates’ phylogenetic trees were created using the packages ape (Paradis and Schliep, 2018) and ggree (Yu et al., 2017). This diversity was also further assessed by rarefaction curves using the package Vegan (Oksanen et al., 2019). Data visualization was further performed using the packages ggplot2 (Wickham, 2016), ggtree (Yu et al., 2017), ggnewscale (Campitelli, 2020), and cowplot (Wilke, 2020).

### Data Availability

All the ASVs obtained were deposited in the NCBI Short Read Archive under BioProject accession number PRJNA682103. Sanger sequences of the individual OTUs (189 OTUs) were deposited in Genebank, submission SUB8892339, sequences MW546078 to MW546266.

## Results

### The Resuscitation Effect of SRpf on Model Bacterial Taxa

**FIGURE 2:**
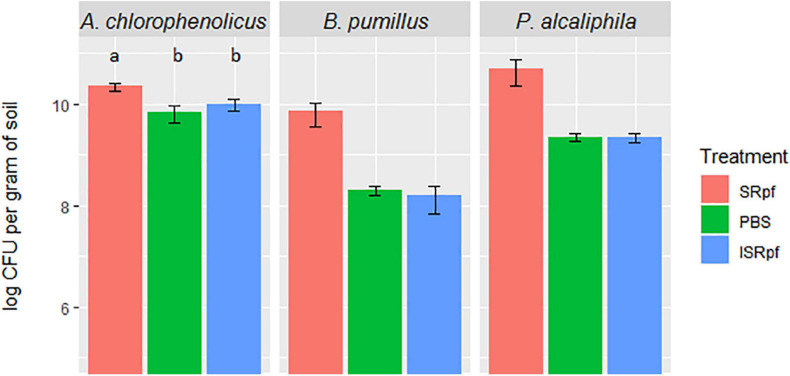
CFUs of each bioaugmented strain present per gram of soil after 1 day of extraction with SRpf, PBS, or ISRpf. *B. pumilus* and *P. alcaliphila* exhibited the highest increase in CFU numbers after extraction with SRpf (more than one order of magnitude versus controls). The number of CFUs for *A. chlorophenolicus* were significantly different among the different treatments (ANOVA, *F* = 105.83, *d.f*.1 = 2, *d.f.*2 = 42, *P* < 0.001). *Post hoc* comparisons (Tukey honest significant differences) show that the *A. chlorophenolicus* CFU numbers for the three conditions significantly differ from each other (*P* < 0.05).

The effectivity of SRpf and the controls (ISRpf and PBS) was first assessed for three strains used individually for the bioaugmentation of soil and incubated there for 1 month. The number of colony-forming units (CFUs) on plates (in each drop) for each of the three bioaugmented strains was higher for the SRpf-treated extractions than for the controls, i.e., ISRpf-treated extractions and PBS (Figure 2). *Bacillus pumilus* and *Pseudomonas alcaliphila* exhibited the highest increase in CFU numbers after extraction with SRpf (more than one order of magnitude versus controls). SRpf was not as effective for *Arthrobacter chlorophenolicus* (non-sporulating, gram-positive bacterium), but the numbers of CFUs were still significantly higher after SRpf-treated extraction than the controls (ANOVA, *F* = 105.83, *d.f.*1 = 2, *d.f.*2 = 42, *P* < 0.001).

### Resuscitation Potential of SRpf on the Whole Community Level

**FIGURE 3:**
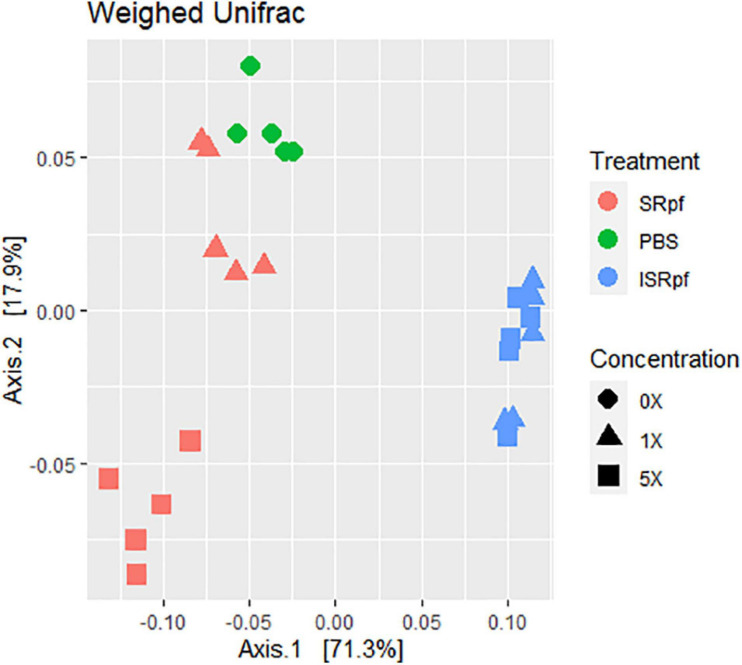
Weighted UniFrac distance ordination method for MDS showing distance between different treatments and concentration levels.

The resuscitation potential of SRpf was further assessed at the level of whole communities by 16S rRNA gene high-throughput amplicon sequencing. Microbial populations from soil responded to both SRpf and ISRpf, but the communities of both SRpf- and ISRpf-treated soil extracts differed significantly (Figure 3; PERMANOVA, 1000 permutations, factor “treatment,” 5 levels, *F* = 38.69, *d.f*.1 = 4, *d.f*.2 = 20, *P* = 0.001). Furthermore, the community composition changed with increasing SRpf concentration, which was not the case for ISRpf, where the effect on the community was similar regardless of the concentration used (Figure 3). There were no significant differences between the treatment’s dispersions (betadisper, factor “treatment,” 5 levels, *F* = 0.6112, *d.f*.1 = 4, *d.f*.2 = 20, *P* = 0.648).

**FIGURE 4:**
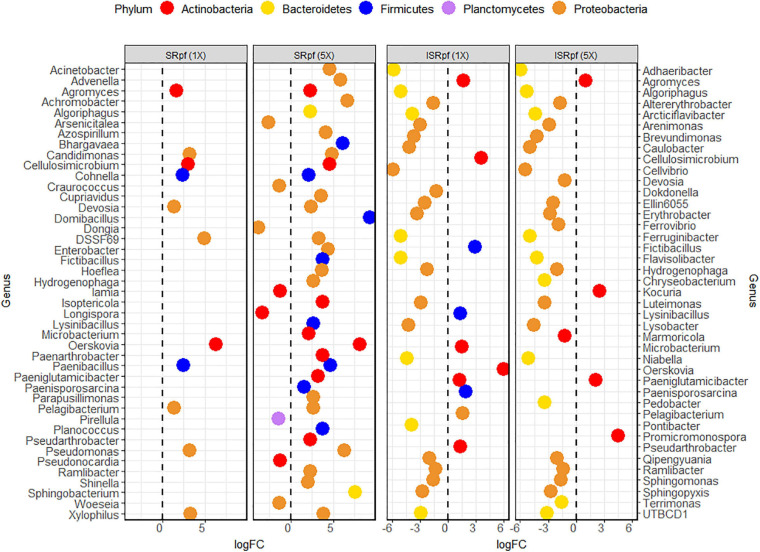
Differential abundance analysis (EdgeR) for the different treatments and their concentration levels. Each treatment and concentration level was compared to the control extraction (extraction with PBS alone).

Figure 4 shows the differential abundance analysis of each treatment and concentration level compared to the PBS extraction. Only the amendment with SRpf resulted in an increase in the abundance of certain genera, namely *Agromyces*, *Cellulosimicrobium*, *Cohnella*, *Candidimonas*, *Devosia*, the undescribed genus DSSF69, *Oerskovia*, *Paenibacillus*, *Pelagibacterium*, *Pseudomonas*, and *Xylophilus*. The number of enriched genera was higher when a higher SRpf concentration was used (Figure 4). The enriched genera spanned several phyla (Figure 4). SRpf increased the abundance of genera distributed widely among Proteobacteria, i.e., representatives from the orders Burkholderiales, Hyphomicrobiales, Enterobacterales, Pseudomonadales, Rhodospirillales, and Sphingomonadales; Firmicutes (class Bacilli); and Bacteroidetes (genera *Algoriphagus* and *Sphingobacterium*). Bacteroidetes from the order Cytophagales and the closely related family Sphingobacteriia were also more abundant after the extraction with a higher concentration of SRpf (García-López et al., 2019).

For ISRpf, there was an increase in the abundance of a few genera, and the abundance of many others remained lower than for the control. Bacteria whose abundance was lower than in PBS belonged mainly to the phyla Bacteroidetes and Proteobacteria, while the genera with higher abundance were mainly those of Firmicutes and Actinobacteria (Figure 4). The abundance of the following genera was increased in both SRpf and ISRpf extractions: *Agromyces*, *Cellulosimicrobium*, *Fictibacillus*, *Lysinibacillus*, *Microbacterium*, *Oerskovia*, *Paeniglutamicibacter*, *Paenisporosarcina*, *Pelagibacterium*, and *Pseudarthrobacter*. Actinomycetes were mainly upregulated after both extractions (SRpf and ISRpf), together with some Firmicutes (*Fictibacillus*, *Lysinibacillus*, and *Paenisporosarcina*, belonging to the class Bacilli) and a Proteobacterium (*Pelagibacterium*). All the Firmicutes whose abundance increased with SRpf or ISRpf belong to the class Bacilli.

**FIGURE 5:**
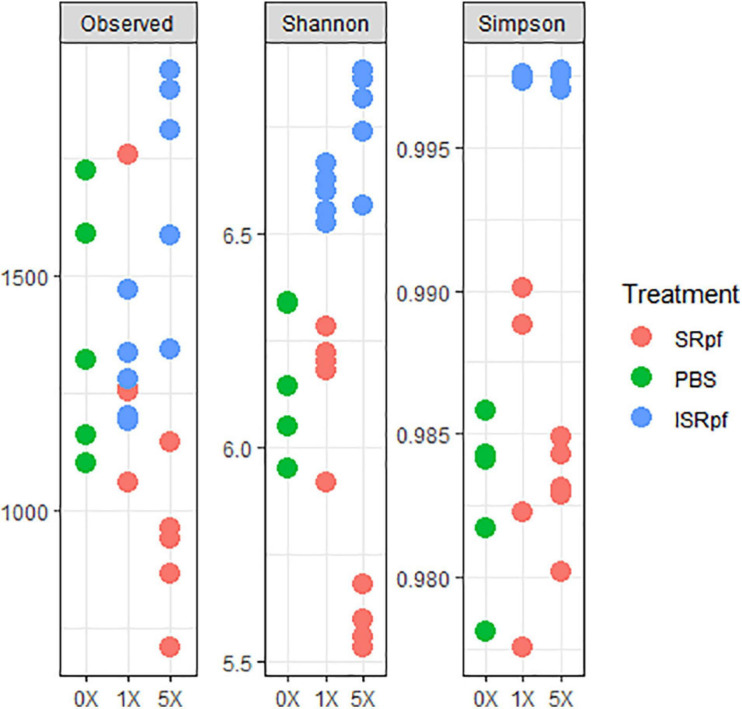
Diversity indexes for each treatment and its concentration level. To obtain the observed number of ASVs, data was rarefied at an even depth of 24522 sequences.

Despite the increased abundance of several genera associated with the use of SRpf, the diversity as suggested by several diversity estimates (Figure 5) was no different from the PBS control or was lower. The number of observed species significantly differed among the five different extraction strategies (extraction with PBS, and with SRpf and ISRPf, both 1× and 5× concentrated; ANOVA, *F* = 8.2054, *d.f.*1 = 4, *d.f.*2 = 20, *P* < 0.001). *Post hoc* comparisons (Tukey honest significant differences) showed that the observed number of ASVs for the 5× SRpf extraction and the 5× ISRpf extraction were significantly different from the PBS extraction (*P* = 0.019 and 0.036, respectively). The 1× supernatant extractions (both active and inactive) did not significantly differ from the PBS extraction (*P* > 0.05).

### Potential of SRpf to Enhance the Diversity of Soil Bacterial Isolates

**FIGURE 6:**
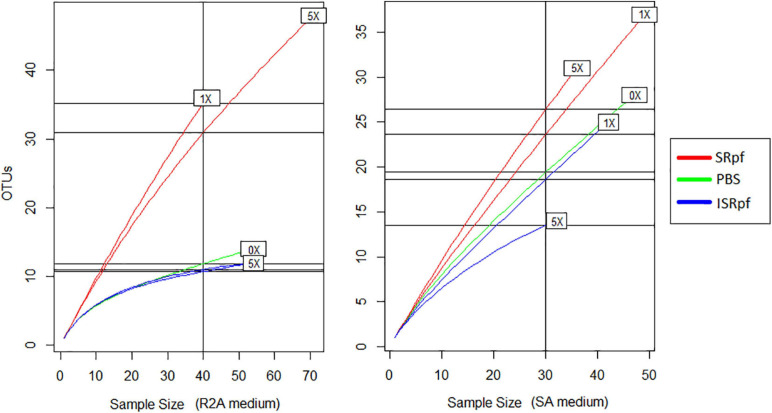
Rarefaction curves of culturable fractions on R2A (left panel) and SA (right panel) for each treatment and concentration.

After 1 day of agitation of soils with SRpf, ISRpf or PBS, soil suspensions were diluted and plated on R2A and SA. In total, 480 isolates were identified based on their 16S rRNA sequence after dereplication with MALDI-TOF MS. Both the abundance and the diversity of the isolates were higher after the use of SRpf compared to both PBS and ISRpf controls, as observed in the rarefaction curves (Figure 6) and the Chao1 estimators (Table 1). In particular, on R2A, the rarefaction curves (Figure 6, left panel) show that 40 isolates belong to 35 different OTUs (SRpf 1×), but only to 15 for the PBS control. In other words, when SRpf was included, more than double the number of OTUs were observed on R2A. Additionally, the diversity differences between SRpf and both ISRpf and PBS controls were more pronounced on R2A than on SA. The diversity differences resulting from the use of 1× and 5× SRpf were not very marked on SA (Figure 6, right panel).

**TABLE 1:** Chao1 estimator for each treatment, concentration, and agar type (SA and R2A).

		Chao1	
Treatment	Concentration	R2A	SA
**SRpf**	1×	130	166
	5×	142	138
**PBS**	0×	65	33
**ISRpf**	1×	22	99
	5×	16	24

*For each medium type, data was rarefied to the treatment with the lowest number of isolates recovered.*

The Chao1 estimator (Table 1) was higher for the isolates retrieved from both media after the SRpf treatment. In R2A, the Chao 1 estimator for 1× and 5× SRpf, respectively, was 130 and 142, while it only reached 22 and 16 for ISRpf (1× and 5×), respectively. Furthermore, the Chao1 estimator was higher for SA than for R2A. More unique colonies (colonies of taxa appearing only once in all the plates, also referred to as singletons) were detected more often for SRpf-treated soils and on SA medium.

**FIGURE 7:**
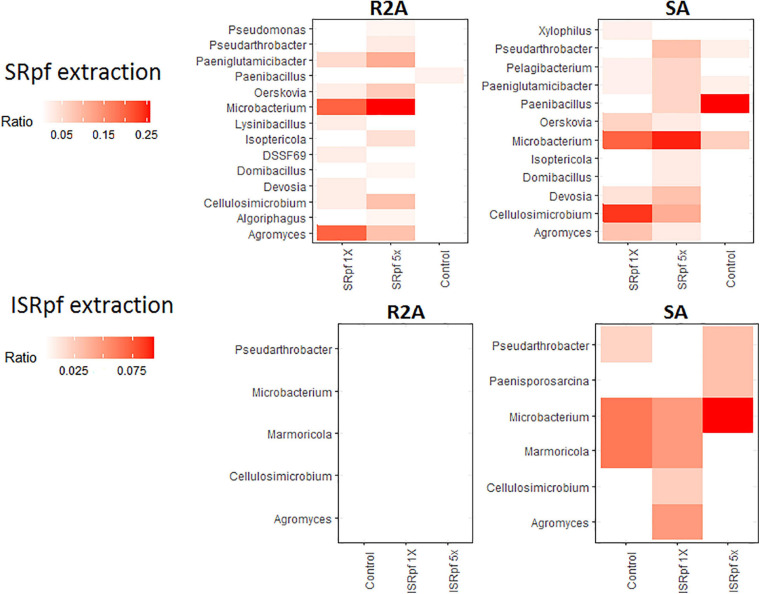
Dominance of each differentially abundant genus from ASV data (Figure 2) on SA or R2A plates, for different extraction strategies (SRpf, upper panels and ISRpf, lower panels). A higher color intensity represents a higher abundance of that genus growing on a specific solid medium. Ratios represent the number of isolates of a particular genus retrieved from a particular condition, divided by all the isolates retrieved from that same specific condition (e.g., from 5× SRpf-treated soils plated on R2A, i.e., the upper left panel, the second column, *Microbacterium* comprised 25% of all the retrieved isolates). Only the genera for which a differential abundance was detected in the ASV data are considered in this figure.

Members of some genera for which an increased abundance was detected in the ASV data (Figure 4) were also more abundant on solid media; Figure 7 shows the dominance of each cultured genus for each specific treatment and the medium used for its cultivation (only the genera for which a differential abundance was detected in ASV data are considered in this figure). Importantly, SRpf (Figure 7, upper panel) cultured more previously uncultured isolates of the genera *Microbacterium*, *Agromyces*, *Paeniglutamicibacter* or *Cellulosimicrobium*, for which an increased abundance was also observed in soil (Figure 4), including a member of the DSSF69 genus, a hitherto uncultured bacterium which was cultured on R2A medium (Figure 7 upper panel).

**FIGURE 8:**
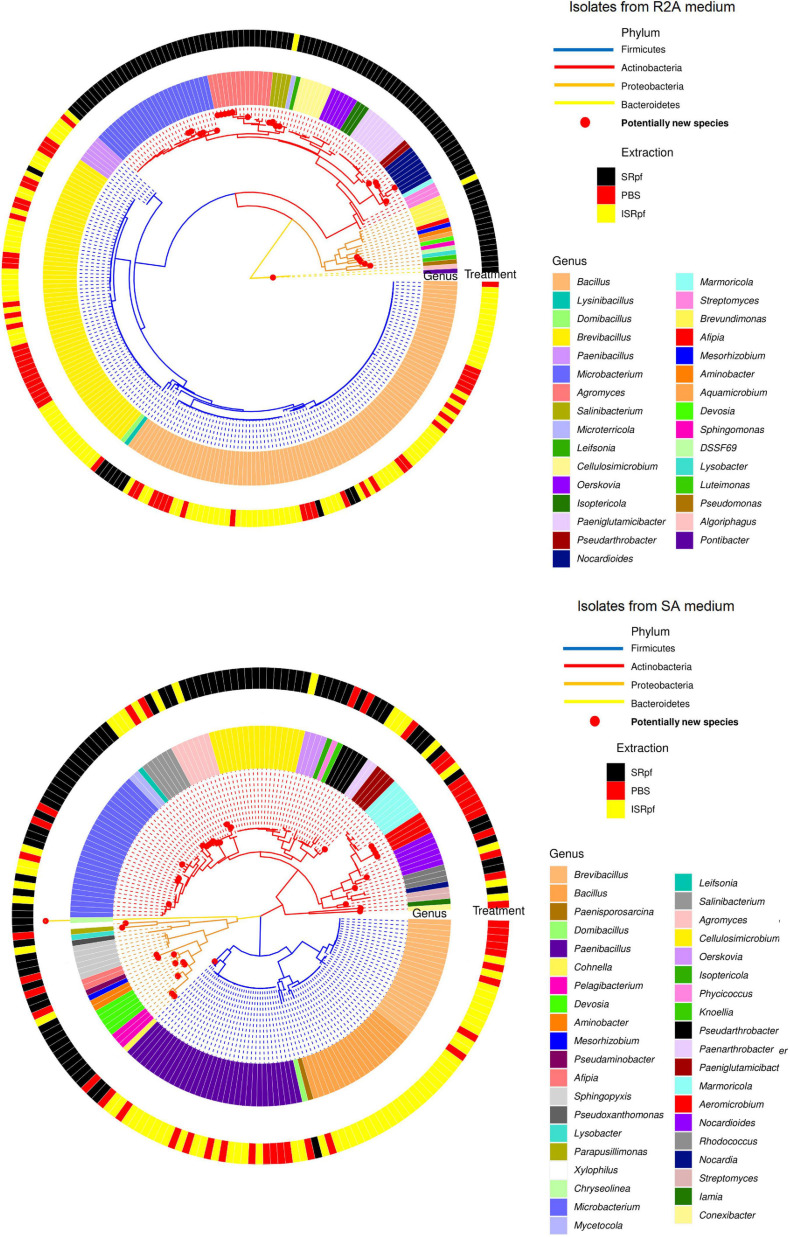
Isolate-based phylogenetic tree of community cultured on R2A medium (upper panel, 274 isolates) and SA medium (lower panel, 206 isolates). Genera are read clockwise starting at 3:00.

Figure 8 shows a phylogenetic tree of the isolates obtained on both R2A and SA (upper and lower panels, respectively) with all the different extractions. SRpf monopolizes the observed diversity on R2A plates (Figure 8, upper panel), which was indicated by previous figures. All the potentially novel bacterial species, i.e., isolates with gene sequence similarity lower than 98.65 (Kim et al., 2014) which grew on R2A, only appeared on the plates after SRpf treatment and totaled 23. In addition to the hitherto undescribed genus DSSF69, SRpf aided the cultivation of potentially novel species of *Agromyces*, *Microbacterium*, *Salinibacterium*, *Nocardioides*, *Lysobacter, Sphingomonas*, *Devosia*, *Pontibacter*, and *Luteimonas* (Figure 8, upper panel). R2A plates of the ISRpf- and PBS-treated soils were dominated by bacilli. Nevertheless, on SA, novel diversity could not be ascribed solely to SRpf. In fact, treatments do not cluster in a specific region of the phylogenetic tree as with R2A, but are more dispersed (Figure 8, lower panel). Similarly to R2A, the genera *Bacillus, Brevibacillus*, and *Paenibacillus* appeared mainly on plates of ISRpf- and PBS-treated soils (Figure 8, lower panel). The total number of potentially novel species on SA plates after SRpf treatment was 12, while after ISRpf and PBS treatments it was 6 and 10, respectively.

## Discussion

Cultivation might have been overlooked recently due to the tremendous amount of information provided by the “omics” technologies (Gutleben et al., 2018), but it is still important, because it is a reliable way to validate these data suggested by the multi-omics technologies, curate databases and develop novel biotechnological capabilities (Gutleben et al., 2018). Previous research shows that SRpf increased the diversity of the communities where it was supplemented (Su et al., 2015, 2018a). Therefore, we hypothesized that the use of SRpf during the extraction of soil bacteria will not only result in an increased diversity of isolated taxa, but also that this higher diversity would translate into more hitherto-uncultured organisms on solid media.

Our experiment was a two-level selection to bring bacteria into culture: SRpf benefits specific community members which then have a bigger chance to be selected by the medium on which they are cultured. The treatment with SRpf induced the replication of specific soil genera distributed among several phyla, as shown by the culture-independent screening of differentially expressed genera (Figure 4). SRpf was effective at increasing the culturability of bacteria with different cell wall structures, as shown by Figure 2. Apparently, the more abundant genera even increased in numbers during the extraction step (28°C for 1 day, agitation at 130 rpm, Figure 4), giving themselves a bigger chance to appear on plates (Figure 7). The idea that only specific genera benefit from SRpf is also supported by the diversity estimates, specifically the number of observed ASVs (Figure 5). Both the number of observed ASVs and the Shannon index go down with increasing SRpf concentration (Figure 5), which suggests an unequal effect of SRpf on each community member (Figures 4, 5). Some organisms increase in number more than others thanks to SRpf, thus their dominance increases, and they may overgrow other less responsive bacteria.

Some of the bacteria for which SRpf had a positive effect in this study responded similarly in previous experiments. These genera include *Pseudomonas* (Jin et al., 2017; Su et al., 2019, 2021), *Bacillus* (Jin et al., 2017; Yu et al., 2020) or *Microbacterium* (Jin et al., 2017), all of which are phylogenetically different and encompass different cell wall structures. At a coarser taxonomic resolution, previous research showed that the phyla Actinobacteria, Proteobacteria and Bacteroidetes increased in abundance after SRpf addition (Su et al., 2015, 2018a, 2019, 2021). Akin to those studies, SRpf proved to be effective at increasing the abundance of representatives of the phyla Proteobacteria, Actinobacteria and Firmicutes in our experimental settings (Figure 4). However, isolates belonging to the phylum Actinobacteria dominated on both media when SRpf was added (Figure 8). The majority of the isolates from Actinobacteria belonged to the order Micrococcales (Figures 4, 8), which is the order to which *M. luteus* (the SRpf source) belongs. Although SRpf increased the abundance among the Proteobacteria (Figure 4), Actinobacteria were more dominant on solid media after SRpf extraction (Figure 8). This suggests that SRpf was most effective at increasing the culturability of bacteria that are most closely related to *M. luteus*. The abundance also increased for some representatives belonging to the phylum Bacteroidetes (Figure 4): The abundance increased for *Algoriphagus* and *Sphingobacterium* (Figure 4, Algoriphagus being successfully cultured on R2A, Figures 7, 8) and representatives from *Pontibacter* and *Chryseolinea* were successfully cultured. These isolates belong to the order Cytophagales and the closely related family Sphingobacteriia (García-López et al., 2019). All of the isolates retrieved from both media belonged to either Bacteroidetes, Proteobacteria, Firmicutes or Actinobacteria.

Importantly, several hitherto uncultured bacteria were isolated in this experiment upon SRpf treatment on both media used for cultivation. For instance, 11 isolates of the most abundant genera growing on R2A plates after SRpf addition, namely *Microbacterium* and *Agromyces* (Figure 7, upper panels), belong to eight potentially novel species (four different *Agromyces* species and four *Microbacterium* species, Figure 8). Other genera whose abundance increased thanks to SRpf addition (Figure 4) and were therefore cultured on media (Figure 7) contained potentially novel species, which is the case for *Xylophilus*, *Devosia* or *Pelagibacterium* (Figure 8). All these genera belong to the phylum Proteobacteria. The genus DSSF69, whose abundance also increased thanks to SRpf addition (Figure 4), was also successfully cultured on R2A (Figure 8). DSSF69 belongs to the order Sphingomonadales, likely to the family Sphingosinicellaceae (Hördt et al., 2020), phylum Proteobacteria. The closest type strains to the DSSF69 isolate by their 16S rRNA sequence similarity according to the EzBioCloud database (56% completeness) are *Sandarakinorhabdus cyanobacteriorum* and *Sphingoaurantiacus capsulatus*, with a similarity of 92.4 and 92.27%, respectively (Yoon et al., 2017). These similarities are much lower than the similarity threshold of 98.65% for considering a novel species (Kim et al., 2014).

In accordance with our hypothesis, plates of SRpf-treated soils harbored more unique isolates than plates after ISRpf or PBS treatment. In this sense, SRpf diminished the cultivation bias, especially on R2A (Figures 7, 8, upper panels), since many genera that were enriched after SRpf addition (Figure 4) were successfully cultivated using solid media (Figure 7, upper panels). ISRpf was not effective at decreasing the cultivation bias (Figure 7, lower panels). The number of isolates retrieved from the different media and extractions (PBS, SRpf, and ISRpf) sufficiently shows the diversity differences between these strategies (Figure 6); in addition, for SRpf treated soils plated on R2A and SA media in general, rarefaction curves were not close to reaching the plateau (Figure 6), which indicates that more isolates being collected from the plates would cause more OTUs to be identified efficiently.

Other isolates with a low 16S rRNA sequence similarity to the described species which were retrieved from plates do not belong to the most abundant genera cultured (Figure 7) or taxa whose abundance significantly increased (Figure 4), but were singletons. On SA, potentially novel species grew regardless of the conditions used during their extraction step, and their growth was not monopolized by SRpf (Figure 8, lower panel). Three of these isolates belong to the phylum Actinobacteria. The first one is an isolate of the family Iamiaceae (genus *Iamia*), a deep-rooting lineage within Actinobacteria with few characterized isolates (Kurahashi et al., 2009). The type strains with the closest similarities according to the EzBioCloud database (Yoon et al., 2017) are *Aquihabitans daechungensis* and *Iamia majanohamensis* (94.27 and 92.35% similarity, respectively). Another Actinobacterium with a less than 98.65% 16S rRNA sequence similarity to the described species is closest to members of the order Solirubrobacterales, *Patulibacter brassicae* (family Patulibacteraceae, 93.73% similarity) and *Paraconexibacter algicola* (family Paraconexibacteraceae, 93.49% identity) (Yoon et al., 2017). The isolate was classified into the genus *Conexibacter* (family Conexibacteraceae), which is also under the order Solirubrobacterales (Figure 8, lower panel). The third isolate is closest to members of the genera *Nocardioides* (*Nocardioides iriomotensis*, 95.66%) and *Marmoricola* (*Marmoricola solisilvae*, 95.16%) of the family Nocardioidaceae (Nesterenko et al., 1985). Even though members from the phylum Bacteroidetes were not amply represented on R2A or SA plates, some genera responded to SRpf addition (Figure 4). An isolate from this phylum that was closest to the genus *Chryseolinea* was isolated from SA after SRpf addition. This genus contains only two described species (Kim et al., 2013), *Chryseolinea serpens* and *C. flava*, the type strains of which have a similarity of 94.06 and 92.18%, respectively, to the isolated bacterium.

The addition of SRpf was less relevant for the cultivation of novel isolates on SA compared to R2A (Figure 8). The SA is a medium containing the soil’s original components: not only minerals, but also metabolites and molecular debris of the whole microbial community. Compounds in SA such as signaling molecules (Bruns et al., 2002, 2003), amino acids (Pinto et al., 2011) or growth factors (Kell and Young, 2000; Kana and Mizrahi, 2010), may already aid the culturability of certain taxa. Because of its oligotrophic nature, it also selects for slow growing bacteria, making it more difficult for certain copiotrophs to overgrow plates. The SA thus more closely resembles *in situ* cultivation strategies, since some of the original metabolites that are present in the soil and are important for bacterial growth are provided by the medium.

It is also worth noting the genera whose abundance increased when either SRpf or ISRpf was added, such as *Agromyces*, *Cellulosimicrobium*, *Oerskovia*, or *Paeniglutamicibacter* (Figure 4). These genera belong to the phylum Actinobacteria. The resuscitation of several members of the phylum Actinobacteria, to which *M. luteus* belongs, is controlled by Rpfs (Mukamolova et al., 2006). These molecules are likely to be distributed among other high GC-content gram-positive bacteria than *M. luteus* (Nikitushkin et al., 2016). Bacteria for which both SRpf and ISRpf were effective could give some clues to Rpf’s mode of action. Three models for Rpf-mediated resuscitation have been proposed (Pinto et al., 2015). In the first model, Rpf triggers resuscitation when it remodels the cell wall of the target cell due to its enzymatic activity. Cells in the viable but non-culturable state (VBNC) can exhibit an increased cross-linking in their peptidoglycan structure and thickened cell walls (Signoretto et al., 2000), so peptidoglycan cleavage by Rpf can help to boost division with a constraining cell wall, especially if it acts upon the peptidoglycan in the septum while replicating (Hett et al., 2007). In the second and third models, Rpf or its cleavage products (for example peptidoglycan fragments), bind to specific receptors on the target cell’s surface (Pinto et al., 2015). Because the presence of peptidoglycan fragments in the environment is a marker for replicating bacteria, they can act as a cue to trigger the resuscitation of dormant bacteria (Shah et al., 2008; Nikitushkin et al., 2013). It is then possible that the bacteria that were higher in abundance after both SRpf and ISRpf additions can point to the existence of specific Rpf receptors (the second Rpf-mediated resuscitation model), which explains their higher abundance in both active and inactive SRpf treatments. The abundance indeed increased for some Actinobacteria after ISRpf addition (Figure 4), but it failed to culture those more abundant genera (Figure 8), as SRpf did. Explaining Rpf’s resuscitation mechanism is beyond the scope of this work and will require further experiments.

In summary, our report shows that SRpf effectively increases the culturability of soil bacteria compared to its inactive form ISRpf (carbon source control) and to the control with PBS alone. Several hitherto-uncultured bacteria were successfully cultured in this study and are awaiting further characterization. This diversity increase after the SRpf treatment, especially on R2A, clearly indicates the potential of SRpf to successfully target more soil diversity and culture hitherto uncultured taxa. In particular some fastidious bacterial clades with the potential of being cultured in a specific medium can be supplemented with SRpf, either to promote their multiplication or resuscitation, before their inoculation and cultivation on any laboratory medium. With this in mind, we propose that SRpf could be included in high-throughput culturing approaches, for example using automated colony-picking systems and under different and extensive cultivation conditions, to allow the growth of different bacteria in the community (Nowrotek et al., 2019). We also show that the SA proved to be highly effective for isolating hitherto-uncultured bacteria. This shows how important it is for some fastidious bacterial species to grow in the presence of metabolites and conditions that most resemble their original environment. The share of uncultured bacteria in the environment remains extremely large, but our tools are also increasing in complexity, automatization and throughput. This can be taken as an incentive for not giving up in this long but rewarding quest of culturing the yet-uncultured.

## Data Availability Statement

All the ASVs obtained were deposited in the NCBI Short Read Archive under BioProject accession number PRJNA682103. Sanger sequences of the individual OTUs (189 OTUs) were deposited in Genebank, submission SUB8892339, sequences MW546078 to MW546266.

## Author Contributions

ML, MS, JSu, and OU planned and designed the research. ML, MS, PJ, and JSa performed the experiments and analyzed the data. ML, MS, and OU wrote the manuscript. All authors contributed to the article and approved the submitted version.

## Conflict of Interest

The authors declare that the research was conducted in the absence of any commercial or financial relationships that could be construed as a potential conflict of interest.

## Footnotes

1 https://baddna.uga.edu/
